# THE IMPACT OF HOME HEALTH CARE UTILIZATION ON SUBSEQUENT HOSPICE USE AMONG MEDICARE BENEFICIARIES

**DOI:** 10.1093/geroni/igad104.0247

**Published:** 2023-12-21

**Authors:** Olga Jarrín, Anum Zafar, Paul Duberstein, Bei Wu, Haiqun Lin, Hyosin Kim, Maria Lopez, S Marie Harvey

**Affiliations:** Rutgers, The State University of New Jersey, New Brunswick, New Jersey, United States; Rutgers, The State University of New Jersey, New Brunswick, New Jersey, United States; Rutgers School of Public Health, Piscataway, New Jersey, United States; New York University, New York City, New York, United States; Rutgers, The State University of New Jersey, Newark, New Jersey, United States; Oregon State University, Corvallis, Oregon, United States; Rutgers, The State University of New Jersey, New Brunswick, New Jersey, United States; Oregon State University, Corvallis, Oregon, United States

## Abstract

Home health care may increase the use of hospice among Medicare beneficiaries by providing improved access to care, enhanced care coordination, and earlier identification of the need for hospice. This study evaluated home health care use during the last three years of life as a predictor of death in the care of hospice for a cohort of 2,169,496 Medicare beneficiaries who died in 2019. The cohort included beneficiaries continuously enrolled in Medicare for 5 or more years, aged 40 and older, and living in the U.S. fifty states, District of Columbia, U.S. Virgin Islands, or Puerto Rico at death. Co-variates included acute hospitalizations during the look-back period, age at death, sex, race/ethnicity, comorbidities, insurance, rural/urban area, and state of residence. Overall, 46% of beneficiaries used home health care during the lookback period, and 62% died at home. Among beneficiaries who used home health care, 59% died in the care of hospice, compared to 45% who never used home health care. Beneficiaries who received home health care at any point during the lookback period were more likely to later die at home (OR 1.10) or in the care of hospice (OR 1.07) compared to their counterparts without a history of using home health care. Additionally, persons with dementia had greater odds of home death (OR 1.21) and death with hospice (OR 1.68) compared to their counterparts without dementia. This study provides strong evidence of the value of home health care as a strategy to improve end-of-life care quality for Medicare beneficiaries.

